# Effectiveness of Inactivated Coronavirus Disease 2019 Vaccine Against Omicron BA.2.2 Infection in Beijing, China, 2022: A Cohabitation Retrospective Cohort Study

**DOI:** 10.3390/v17010031

**Published:** 2024-12-28

**Authors:** Shuaibing Dong, Ying Sun, Zhaomin Feng, Yi Tian, Lei Jia, Xiaoli Wang, Quanyi Wang, Daitao Zhang, Peng Yang

**Affiliations:** 1 Beijing Center for Disease Prevention and Control, Beijing 100013, China; dongshuaibing158@126.com (S.D.); sun1ying2@163.com (Y.S.); fengzhaomin214@163.com (Z.F.); wangxiaoli198215@163.com (X.W.); bjcdcxm@126.com (Q.W.); 2 Beijing Research Center for Respiratory Infectious Diseases, Beijing 100013, China; 3 School of Public Health, Capital Medical University, Beijing 100069, China

**Keywords:** Omicron BA.2.2, inactivated COVID-19 vaccines, vaccine effectiveness, COVID-19 pandemic, booster immunization, China

## Abstract

The present study aimed to evaluate the vaccine effectiveness (VE) of different doses of an inactivated coronavirus disease 2019 (COVID-19) vaccine against Omicron BA.2.2 infection in Beijing, China, 2022. Based on data from a previous cohabitation retrospective cohort of COVID-19 outbreak in Beijing, China, 2022, the cohabitating contacts of individuals with BA.2.2 infection were followed up. Using a log-binomial regression model in which the unvaccinated group as the control group, the risk ratios of different doses of inactivated vaccine in terms of preventing SARS-CoV-2 infection, symptoms of COVID-19, and pneumonia were calculated, and the protective effect of the vaccine was estimated. The Kruskal–Wallis rank-sum test was used to compare the effect of vaccination on the viral load of infected patients. From April to June 2022, a total of 2259 cohabiting close contacts of 1308 patients with SARS-CoV-2 infection aged ≥3 years were included. Of the included close contacts, 737 (32.63%) were positive for SARS-CoV-2 during the isolation period: 140 (19.00%) were infected but asymptomatic, 525 (71.23%) had mild infection, and 72 (9.77%) had pneumonia. There were no cases of severe or critical infection or death. The VE of the primary in preventing BA.2.2 infection, symptoms, and pneumonia was 37.35% (95% CI: 24.00–48.35), 42.36% (95% CI: 28.41–53.60), and 48.35% (95% CI: −5.34–74.67), respectively. The VE of the booster shot in preventing SARS-CoV-2 infection, symptoms, and pneumonia was 37.08% (95% CI: 24.29–47.70), 44.38% (95% CI: 31.45–54.87), and 61.46% (95% CI: 29.79–78.85), respectively. Six months after the booster vaccination, the VE of the booster in terms of preventing SARS-CoV-2 remained above 46%, and its VE in terms of the prevention of pneumonia remained above 72%. In the unvaccinated group, the Ct values of the N gene and ORFlab gene (represented by the median value and Q1 and Q3 in parentheses) were 26.45 (21.09, 31.61) and 28.06 (22.21, 32.06), respectively. There was no significant difference in the median value of either gene between the unvaccinated group, the partial group [25.81 (19.91, 31.78) and 26.98 (21.63, 31.17)], the primary group [28.79 (22.08, 32.34) and 29.30 (23.81, 33.86)], and the booster group [26.23 (21.66, 31.46) and 27.73 (23.38, 32.52)] (*p* > 0.05). Inactivated COVID-19 vaccines provided a certain level protection from infection and symptoms, very good protection against pneumonia, and it still has a modest protective effect at 6 months after vaccination. Booster doses are necessary to provide strongest protection. However, irrespective of their vaccination status, individuals with COVID-19 have a similar viral load.

## 1. Introduction

COVID-19 is an infectious disease caused by the severe acute respiratory syndrome coronavirus-2 (SARS-CoV-2). The COVID-19 pandemic has had a serious impact on global health and economic development [1]. Vaccination against COVID-19 is still considered to be one of the most effective ways to manage the COVID-19 epidemic in the long term [2,3]. As of 31 December 2023, a total of 13.64 billion doses of COVID-19 vaccine had been administered worldwide: more than 70.0% of the world population has received at least one dose, 67.0% have completed the primary, and 32.0% have received booster immunization [4]. Since the beginning of 2021, two similar composition COVID-19 inactivated vaccines, CoronaVac and BBIBP-CorV, have been widely used for vaccination at the national level in China [5]. Although two doses of inactivated COVID-19 vaccine can provide effective protection from COVID-19, especially in severe cases [5,6], the effectiveness of the vaccine rapidly diminishes with time [7]. As a result, in November 2021, the National Health Commission of China launched a drive for administering a third dose of intensive vaccination with homologous or heterologous vaccines for adults ≥ 18 years of age in whom at least 6 months have elapsed since the primary series in mainland China. Furthermore, in order to minimize the morbidity and mortality of COVID-19, China has implemented strict non-pharmaceutical interventions to reduce the spread of human infection [8].

The Omicron BA.2 variant was detected for the first time in the United States on 27 November 2021. Because of its faster transmission speed, stronger immune escape ability, and lower degree of disease severity than Delta and Omicron BA.1, OmicronBA.2 has rapidly spread all over the world and has become the dominant strain. From February to July 2022, large-scale Omicron BA.2 outbreaks occurred in the Beijing, Shanghai, Guangdong, and Hong Kong Special Administrative Region of China [9,10,11]. Hong Kong, China, was the first to evaluate the protective effect of BNT162b2 and CoronaVac vaccines on Omicron BA.2 infection through a population-based observational study. The results showed that three doses of either vaccine provided a very high level of protection against severe illness and death (97.9% [97.3–98.4]) [12]. Some Chinese scholars have also analyzed the protective effect of COVID-19 inactivated vaccines on Omicron BA.2 infection through a retrospective cohort study, however, all the included cases occurred during the Omicron BA.2 epidemic, during which all close contacts were followed up irrespective of whether they were cohabitating with the infected individuals [13,14]. There are chances of infection with other variants during the Omicron BA.2 epidemic, and the degree of contact may differ between close contacts. Both these factors may affect the protective ability of the vaccine. In fact, previous studies have found that contact within families is the main route of spread of COVID-19 [15,16]. At present, there are few reports on the protective effect of the COVID-19 vaccine that use a cohabitation retrospective cohort design. Therefore, here, we have conducted a retrospective cohort study on the cohabitating close contacts of SARS-CoV-2-infected individuals. The Omicron BA.2.2 variant was identified by whole-genome sequencing, the close contacts were followed up to evaluate the effectiveness of different doses of a novel coronavirus -inactivated vaccine in preventing SARS-CoV-2 infection, COVID-19 symptoms, and COVID-19 pneumonia.

## 2. Methods

### 2.1. Data Source and Ethical Considerations

Data regarding the close contacts of previously recorded individuals with COVID-19 were obtained from the National Notifiable Disease Report System, and on-the-spot epidemiological investigation. The data gathered included personal basic information, the date of last exposure, and the severity of COVID-19. Vaccine information was obtained from the Immunization Information System and included vaccination dose, vaccination time, and vaccine type. Children under 3 years old are not eligible for the COVID-19 vaccine, so this age group was not included in the study.

### 2.2. Definitions

Close contacts were defined as those individuals who were in close contact with SARS-CoV-2-infected individuals 4 days before the onset of symptoms or 4 days before sampling in asymptomatic cases, did not protect themselves efficaciously. In this study, only close contacts with high exposure risk of cohabiting close contacts.

SARS-CoV-2 infection, symptomatic COVID-19, and COVID-19 pneumonia were diagnosed according to China’s Diagnosis and Treatment Guidelines for COVID-19 Patients [17].

The booster immunity group included individuals who had received three doses of the inactivated COVID-19 vaccine before their last exposure to SARS-CoV-2, with the third dose administered at least 7 days before last exposure [18]. The primary group included individuals who had received two doses of the inactivated COVID-19 vaccine before their last exposure to SARS-CoV-2, with the second dose administered at least 14 days before last exposure. The partial group included individuals who had received only one dose of the inactivated COVID-19 vaccine before their last exposure to SARS-CoV-2, or had received a second dose less than 14 days after the first vaccination. The unvaccinated group included individuals who had not received the COVID-19 vaccine before their last exposure to SARS-CoV-2.

### 2.3. Detection Methods

A previously described SARS-CoV-2 nucleic acid detection method was used [17]. Briefly, throat swab, nasal swab, or nasopharynx swab samples of infected patients were obtained and used to extract nucleic acid by the magnetic bead method. Nucleic acid was detected by real-time fluorescence RT-PCR amplification of two target genes of the SARS-CoV-2 genome, namely, the open reading frame ORF 1ab gene and the N gene. The threshold line was manually adjusted to read the Ct values of the two target genes. Gene Ct value was defined as the number of cycles required for the fluorescence signal to reach the set threshold, and this can indirectly reflect the viral load. Because the Ct value is a dynamic variable, the minimum N gene value in cases of double-gene-positive infection and the corresponding ORFlab gene value were analyzed. The SARS-CoV-2 genome sequences have been performed on randomly selected strains. SARS-CoV-2 whole-genome capture kit (China Micro Future Science and Technology Co., Ltd., No. 1 Longyu Middle Street, Changping District, Beijing, China) was used for cDNA synthesis and multiple PCR amplification according to the kit instructions. The DNA library was constructed by using the Illumina DNA Preparation Kit based on the manufacturer’s instructions, and double-terminal sequencing was carried out on the Illumina Mini Seq sequencing technology platform. The data were assembled with the CLC Work Bench software (version 21) (QIAGEN, Germany). A SARS-CoV-2 gene evolution tree was constructed to carry out gene evolution analysis.

### 2.4. Statistical Analysis

Count data are presented as the constituent ratio. For data with a skewed distribution, median and quartile range are used to describe the concentration and discreteness trend. The χ^2^ test was used to compare categorical data. Using the log-binomial regression model, with the unvaccinated group as the control group and different clinical outcomes as the observation index, the adjusted risk ratios (aRRs) for incomplete, basic, and enhanced immunization for the prevention of infection, symptom, and pneumonia were calculated. In addition, the protective effect of the vaccine was estimated by using the formula aVE = (1 − aRR) × 100%, where aVE represents the vaccine effectiveness. The effect of vaccination on viral load was compared using the Kruskal–Wallis rank–sum test. We used the SAS9.4 software for statistical analysis and the R4.2.0 software for drawing graphs. For bilateral tests, the significance level (α) was set at 0.05.

## 3. Results

### 3.1. Demographic Characteristics of the Participants

From April to June 2022, a total of 2259 cohabiting close contacts of 1308 patients with SARS-CoV-2 infection aged ≥ 3 years were included. The cohort included 1264 males (55.95%). Furthermore, 1749 (77.42%) were 18–59 years old and 288 (12.75%) were over 60 years old. With regard to vaccination status, 140 participants (6.20%) were not vaccinated, 68 (3.01%) were partially vaccinated, 560 (24.79%) had received the primary vaccination, and 1491 (66.00%) had received homologous booster immunization. In the 2119 vaccinated individuals, the interval between the final dose and exposure was less than 3 months in 294 (13.01%) and ≥6 months in 1004 (44.44%) (Table 1).

### 3.2. SARS-CoV-2 Infection

**Figure 1:**
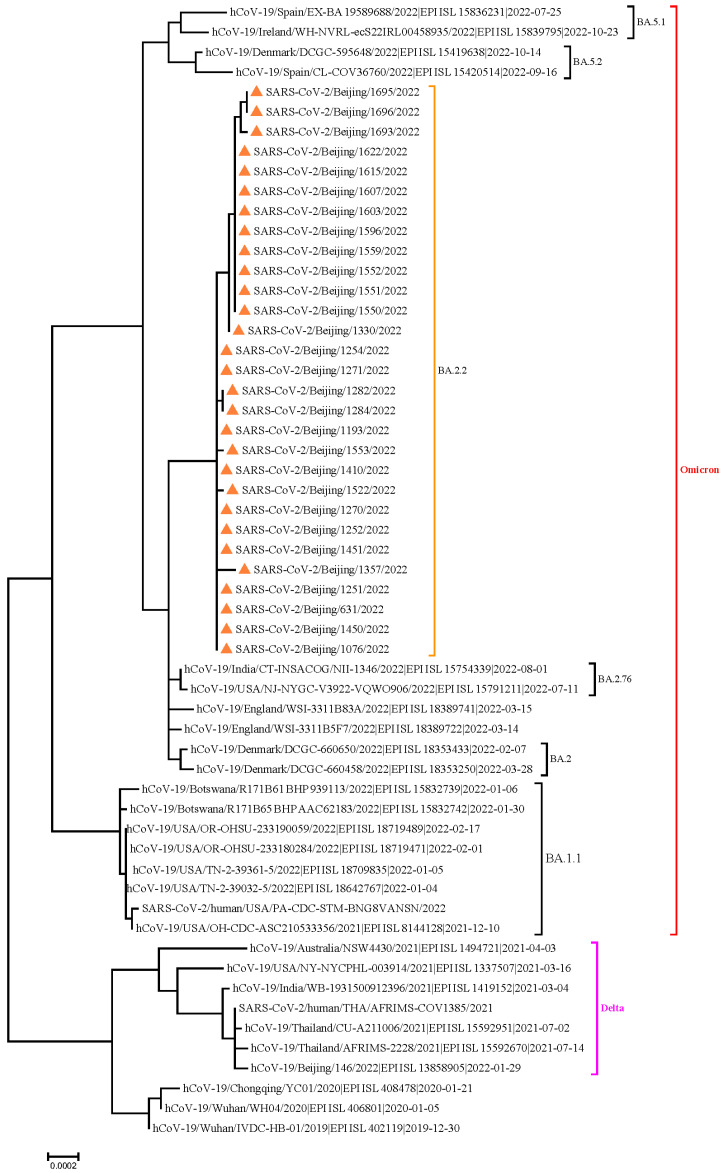
Results of phylogenetic tree analysis of whole genome sequencing all of the SARS-CoV-2 OmicronBA.2.2 variant.

Of the 2259 cohabiting close contacts, 737 (32.63%) were positive for SARS-CoV-2 during the isolation period: 140 (19.00%) had asymptomatic infection; 525 (71.23%), mild infection; and 72 (9.77%), pneumonia. There were no cases of severe or critical infection or death. Whole-genome sequencing of SARS-CoV-2 revealed that all the infection was caused by an Omicron BA.2.2 variant (Figure 1).

### 3.3. Vaccine Effectiveness

Table 2 depicts the protective effect of the inactivated vaccine against three outcomes (SARS-CoV-2 infection, COVID-19 symptom, and COVID-19 pneumonia). With the unvaccinated group as the control group, the results were adjusted for sex and age. According to the adjusted results, the VE of the primary in preventing SARS-CoV-2 infection, symptoms, and pneumonia was 37.35% (95% CI: 24.00–48.35), 42.36% (95% CI: 28.41–53.60), and 48.35% (95% CI: −5.34 to 74.67), respectively. Furthermore, the VE of the booster for the prevention of SARS-CoV-2 infection, symptoms, and pneumonia was 37.08% (95% CI: 24.29–47.70), 44.38% (95% CI: 31.45–54.87), and 61.46% (95% CI: 29.79–78.85), respectively. Six months later, the VE of three doses in terms of preventing SARS-CoV-2 remained above 46%, and the VE in terms of preventing pneumonia remained above 72%.

When the data were stratified by sex, the VE in terms of SARS-CoV-2 infection, symptoms, and pneumonia was 50.50%, 58.07%, and 63.04%, respectively, in males, and 41.84%, 48.15%, and 62.79%, respectively, in females. When the data were stratified by age, it was found that the age group 18–59 years and in the elderly, the VE in terms of preventing pneumonia reached 71.58% (95% CI: 21.56–89.70) and 66.58% (95% CI: 29.30–84.21), respectively (Table 2).

### 3.4. Effect of the Vaccine on COVID-19 Virus Load

**Figure 2:**
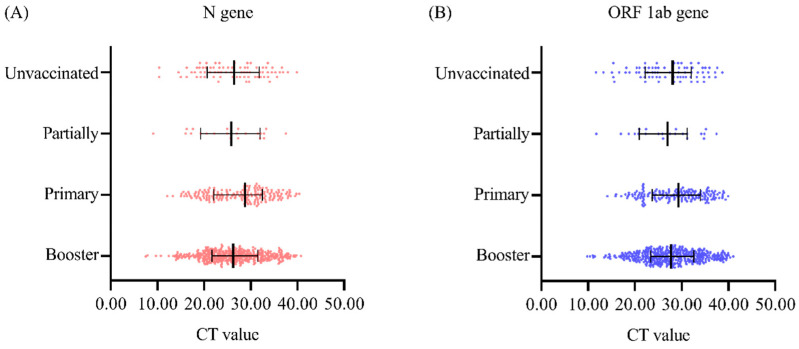
Comparison of nucleic acid Ct values in individuals with SARS-CoV-2 according to vaccination status (**A**) Ct value of the N gene. (**B**) Ct value of the ORF1ab gene.

The Ct values of the N gene in the unvaccinated group, the partial group, the primary group, and the booster group, represented as M (median) (Q1, Q3), were 26.45 (21.09, 31.61), 25.81 (19.91, 31.78), 28.79 (22.08, 32.34), and 26.23 (21.66, 31.46), respectively, and there was no significant difference between the groups (H = 5.70, *p* = 0.127) (Figure 2A). The Ct values of the ORFlab gene in the four groups were 28.06 (22.21, 32.06), 26.98 (21.63, 31.17), 29.30 (23.81, 33.86), and 27.73 (23.38, 32.52), respectively, and there was no significant difference among the four groups (H = 4.95, *p* = 0.176) (Figure 2B).

## 4. Discussion

This study is a real-world, retrospective, cohort study that analyzes the protective effect of an inactivated COVID-19 vaccine in 2259 cohabiting close contacts (age ≥ 3 years) of SARS-CoV-2-infected individuals against Omicron BA.2.2 infection, symptoms, and pneumonia. Because cohabitating family members have more frequent and longer periods of unprotected contact with members infected with SARS-CoV-2, the risk of infection is higher. These dynamics are representative of the natural movement of and communication between individuals who do not undertake specific preventive measures and are, therefore, more reliable than retrospective cohort studies and observational studies based on all close contacts.

The results showed that the protective efficiency of the primary and booster immunization for the prevention of SARS-CoV-2 infection was more than 37%. This is significantly higher than that reported by the national-level retrospective cohort study by Tang et al., who reported inactivated coronavirus disease 2019 vaccine a protective efficiency of 17% (13–21%) and 22% (18–25%) for the primary and booster immunization, respectively [13]. The protective efficiency is also higher than the efficiency of 28.6% (11.6–35.0%) reported in a study from Guangdong on the effect of booster vaccination in preventing Omicron BA.2 infection [14]. The protective efficiency reported here is also higher than that of the case-control study Omicron BA.2 in Shanghai, which was 4.4% (1.9–6.8%) and 18.0% (17.0–18.9%) [19]. The protective efficiency of the booster vaccination in preventing the symptoms of COVID-19 was 44.38% (31.45–54.87%), and the efficiency for the prevention of pneumonia associated with COVID-19 was 61.48% (29.79–78.75%). These values were higher than those reported by a study in Hong Kong which showed that the effectiveness of booster immunization against the symptoms of and pneumonia associated with the Omicron BA.2 COVID-19 variant was 35.7% (22.1–47.3%) and 46.9% (29.6–60.6%), respectively, in participants aged 29–59 years and ≥60 years [12]. The value determined here was also higher than that reported in the Guangdong study mentioned earlier, in which the efficiency of booster immunization in preventing the symptoms of SARS-CoV-2 Omicron BA.2 infection was 39.6% (30.0–47.9%) [14].

In this study, in individuals over 60 years of age, the effect of the booster vaccination was significantly better than that of partial vaccination; this is consistent with the results of most previous studies [12,14,20]. Therefore, based on the present and previous findings, it is strongly recommended that the elderly be vaccinated of booster as much as possible in the absence of contraindications for vaccination. However, in those aged 18–59 years, vaccination with the primary was found to be slightly better than booster immunization.

This study showed that VE was slightly higher in males than in females, as reported by previous studies [13,14]. Considering the effect of the interval between the final dose of vaccination and exposure on the protective effect of the vaccine, it was found that irrespective of the time interval (<3 months, 3–6 months, or >6 months), the protective effect of the primary with booster immunization was better than that of partial vaccination in preventing infection, symptoms, and pneumonia. However, the protective effect of the booster began to weaken after 3 months, which was consistent with the results of Huang et al. [19]. Despite this, a certain level of protective effect was observed even after 6 months; in particular, the protective effect against COVID-19-associated pneumonia remained at 72.95% (38.66–88.07%). Thus, the inactivated COVID-19 vaccines booster immunization provided very good protection against COVID-19 pneumonia.

Some studies have shown that the N gene is more sensitive than the ORF1ab gene for the detection and diagnosis of COVID-19, and PCR amplification of these genes has indicated that the viral load of most patients first tends to decrease before it increases during infection [21]. Therefore, in this study, the minimum N gene value of infected patients, along with the corresponding ORFlab gene value, was used for analysis. There was no significant difference in the median Ct value of both genes between the partial group, primary, and the booster groups. Accordingly, the results of several articles show that SARS-CoV-2-infected individuals have similar viral loads in their body regardless of whether they are vaccinated with the COVID-19 vaccine [22]. It may be that inactivated COVID-19 vaccines are not effective in generating mucosal immunity, thus demonstrating limited ability to prevent SARS-CoV-2 infection and reduce viral load, but vaccines still have a significant effect in preventing COVID-19 pneumonia, severe, and death [13,23].

This study is limited by its retrospective nature. Moreover, the inclusion of only co-resident limited the number of individuals who could be recruited. Therefore, the sample size was small. Furthermore, as demonstrated by the results of stratified analysis, the 95% confidence interval of VE was large, and there were no cases of severe or critical infection or death among cohabitating close contacts. Therefore, it was impossible to evaluate the protective effect of the inactivated vaccine against severe or critical infection or death. In addition, when the effect of vaccination on viral load was analyzed, it was found that vaccination status did not affect viral load. The reasons for these trends are unclear, and further investigation at later stages would be useful to analyze the possible influencing factors.

## 5. Conclusions

In this study, based on an analysis of 2259 individuals aged ≥3 years living in close contact with SARS-CoV-2-infected persons, it was found that the inactivated COVID-19 vaccines provided a certain level protection from infection and symptoms, very good protection against pneumonia. In particular, it is recommended that the elderly be vaccinated of booster as much as possible in the absence of contraindications for vaccination. As the protective effect of the vaccine was observed even 6 months after vaccination, inactivated vaccines may still be a promising option for potential pandemic situations in the future.

## Figures and Tables

**Table 1:** Demographic and COVID-19 characteristics in the cohort of close contacts of SARS-CoV-2-infected individuals.

Variable	None (n = 140)	Partial (n = 68)	Primary (n = 560)	Booster (n = 1491)	Total (n = 2259)	χ^2^ Value	*p* Value
Sex						3.518	0.318
Male	68 (48.57)	40 (58.82)	318 (56.79)	838 (56.2)	1264 (55.95)		
Female	72 (51.43)	28 (41.18)	242 (43.21)	653 (43.8)	995 (44.05)		
Age group (years)						586.457	<0.001
3–17	35 (25)	10 (14.71)	177 (31.61)	0 (0)	222 (9.83)		
18–59	55 (39.29)	51 (75)	337 (60.18)	1306 (87.59)	1749 (77.42)		
≥60	50 (35.71)	7 (10.29)	46 (8.21)	185 (12.41)	288 (12.75)		
Interval between last dose and exposure (months)						2358.972	<0.001
≤3	-	21 (30.88)	49 (8.75)	224 (15.02)	294 (13.01)		
3–6	-	15 (22.06)	159 (28.39)	647 (43.39)	821 (36.34)		
≥6	-	32 (47.06)	352 (62.86)	620 (41.58)	1004 (44.44)		
Infection						41.458	<0.001
Yes	80 (57.14)	23 (33.82)	179 (31.96)	455 (30.52)	737 (32.63)		
No	60 (42.86)	45 (66.18)	381 (68.04)	1036 (69.48)	1522 (67.37)		
Symptoms						51.904	<0.001
Yes	73 (52.14)	16 (23.53)	148 (26.43)	360 (24.14)	597 (26.43)		
No	67 (47.86)	52 (76.47)	412 (73.57)	1131 (75.86)	1662 (73.57)		
Pneumonia						22.499	<0.001
Yes	14 (10)	2 (2.94)	16 (2.86)	40 (2.68)	72 (3.19)		
No	126 (90)	66 (97.06)	544 (97.14)	1451 (97.32)	2187 (96.81)		
Severe infection						-	-
Yes	0 (0)	0 (0)	0 (0)	0 (0)	0 (0)		
No	140 (100)	68 (100)	560 (100)	1491 (100)	2259 (100)		

Data are shown as n (%).

**Table 2:** Effectiveness of the inactivated COVID-19 vaccine against SARS-CoV-2 infection, symptoms, and pneumonia.

Variable	Vaccination	Infection		Symptoms		Pneumonia	
		n/N (%)	aVE (95% CI)	n/N (%)	aVE (95% CI)	n/N (%)	aVE (95% CI)
Total	None	80/140 (57.14)	Ref	73/140 (52.14)	Ref	14/140 (10.00)	Ref
	Partial	23/68 (33.82)	30.36 (−0.61–51.80)	16/68 (23.53)	46.61 (15.26–66.36)	2/68 (2.94)	55.73 (−87.17–89.53)
	Primary	179/560 (31.96)	37.35 (24.00–48.35)	148/560 (26.43)	42.36 (28.41–53.60)	16/560 (2.86)	48.35 (−5.34–74.67)
	Booster	455/1491 (30.52)	37.08 (24.29–47.70)	360/1491 (24.14)	44.38 (31.45–54.87)	40/1491 (2.68)	61.46 (29.79–78.85)
Sex							
Male	None	40/68 (58.82)	Ref	36/68 (52.94)	Ref	9/68 (13.24)	Ref
	Partial	12/40 (30.00)	49.00 (14.78–69.48)	9/40 (22.50)	57.50 (21.21–77.07)	2/40 (5.00)	31.93 (−219.74–85.51)
	Primary	97/318 (30.50)	48.14 (32.82–59.98)	79/318 (24.84)	53.07 (37.00–65.05)	12/318 (3.77)	42.92 (−42.15–77.08)
	Booster	244/838 (29.12)	50.50 (38.00–60.48)	186/838 (22.20)	58.07 (45.76–67.59)	24/838 (2.86)	63.04 (17.67–83.41)
Female	None	40/72 (55.56)	Ref	37/72 (51.39)	Ref	5/72 (6.94)	Ref
	Partial	11/28 (39.29)	29.29 (−17.14–57.31)	7/28 (25.00)	51.35 (4.00–75.35)	0/28 (0)	NA
	Primary	82/242 (33.88)	39.01 (19.99–53.51)	69/242 (28.51)	44.52 (25.07–58.92)	4/242 (1.65)	63.23 (−43.89–90.61)
	Booster	211/653 (32.31)	41.84 (26.46–54.00)	174/653 (26.65)	48.15 (32.87–59.95)	16/653 (2.45)	62.79 (−9.89–87.40)
Age group (years)							
3–17	None	14/35 (40.00)	Ref	11/35 (31.43)	Ref	0/35 (0)	Ref
	Partial	5/10 (50.00)	−21.07 (−236.93–56.49)	2/10 (20.00)	NA	2/10 (20.00)	NA
	Primary	74/177 (41.81)	−2.42 (−81.65–42.25)	66/177 (37.29)	−17.56 (−122.97–38.02)	2/177 (1.13)	NA
18–59	None	34/55 (61.82)	Ref	32/55 (58.18)	Ref	4/55 (7.27)	Ref
	Partial	13/51 (25.49)	57.64 (29.34–74.61)	10/51 (19.61)	65.21 (36.85–80.84)	0/51 (0)	NA
	Primary	84/337 (24.93)	58.90 (45.81–68.82)	66/337 (19.58)	65.16 (52.57–74.4)	6/337 (1.78)	75.52 (15.98–92.87)
	Booster	386/1306 (29.56)	51.61 (39.63–61.21)	302/1306 (23.12)	59.28 (48.21–67.99)	27/1306 (2.07)	71.58 (21.56–89.70)
≥60	None	32/50 (64.00)	Ref	30/50 (60.00)	Ref	10/50 (20.00)	Ref
	Partial	5/7 (71.43)	−9.35 (−80.84–33.88)	4/7 (57.14)	9.81 (−76.66–53.95)	0/7 (0)	NA
	Primary	21/46 (45.65)	29.69 (−2.31– 51.68)	16/46 (34.78)	41.71 (8.44–62.89)	8/46 (17.39)	12.14 (−99.06– 61.22)
	Booster	69/185 (37.3)	42.31 (23.9–56.26)	58/185 (31.35)	48.26 (29.73–61.90)	13/185 (7.03)	66.58 (29.30–84.21)
Interval between last dose and exposure (months)							
	None	80/140 (57.14)	Ref	73/140 (52.14)	Ref	14/140 (10.00)	Ref
≤3	Partial	10/21 (47.62)	15.76 (−62.76–56.40)	7/21 (33.33)	35.73 (−39.74–70.44)	1/21 (4.76)	NA
	Primary	14/49 (28.57)	44.82 (1.59–69.06)	12/49 (24.49)	48.40 (3.89–72.3)	2/49 (4.08)	7.23 (−327.56–79.87)
	Booster	54/224 (24.11)	58.64 (40.14–71.42)	42/224 (18.75)	64.87 (47.43–76.52)	5/224 (2.23)	75.33 (29.49–91.37)
3–6	Partial	7/15 (46.67)	12.27 (−94.32–60.39)	4/15 (26.67)	45.56 (−52.00–80.51)	1/15 (6.67)	NA
	Primary	65/159 (40.88)	25.72 (−7.01–48.44)	57/159 (35.85)	29.60 (−3.70–52.21)	3/159 (1.89)	43.11 (−114.40–84.90)
	Booster	208/647 (32.15)	45.45 (27.28–59.07)	168/647 (25.97)	52.23 (35.30–64.74)	22/647 (3.40)	62.83 (24.98–81.58)
≥6	Partial	6/32 (18.75)	66.82 (21.57–85.97)	5/32 (15.63)	71.49 (27.32–88.81)	0/32(0)	NA
	Primary	100/352 (28.41)	43.53 (22.35–58.93)	79/352 (22.44)	51.19 (31.13–65.40)	11/352 (3.13)	41.14 (−40.68–75.37)
	Booster	193/620 (31.13)	46.28 (27.70–60.09)	150/620 (24.19)	54.64 (37.77–66.94)	13/620 (2.10)	72.95 (38.66–88.07)

## Data Availability

The data are available upon reasonable request to the corresponding authors.
